# Transcriptional, Behavioral and Biochemical Profiling in the 3xTg-AD Mouse Model Reveals a Specific Signature of Amyloid Deposition and Functional Decline in Alzheimer’s Disease

**DOI:** 10.3389/fnins.2020.602642

**Published:** 2020-12-15

**Authors:** Wencheng Yin, Navei Cerda-Hernández, Atahualpa Castillo-Morales, Mayra L. Ruiz-Tejada-Segura, Jimena Monzón-Sandoval, Perla Moreno-Castilla, Rodrigo Pérez-Ortega, Federico Bermudez-Rattoni, Araxi O. Urrutia, Humberto Gutiérrez

**Affiliations:** ^1^Centre for Computational Biology, University of Birmingham, Birmingham, United Kingdom; ^2^Department of Biology and Biochemistry, Milner Centre for Evolution, University of Bath, Bath, United Kingdom; ^3^Dementia Research Institute, Cardiff University, Cardiff, United Kingdom; ^4^National Institute on Aging, National Institutes of Health, Baltimore, MD, United States; ^5^Departamento de Neurociencias, Instituto de Fisiología Celular UNAM, Ciudad de México, México City, Mexico; ^6^Instituto de Ecología UNAM, Ciudad de México, México City, Mexico; ^7^Instituto Nacional de Medicina Genómica, Ciudad de México, México City, Mexico

**Keywords:** beta amyloid deposits, transcriptome, memory decline, neurodegeneration, Alzheimer’s, dementia

## Abstract

Alzheimer’s disease (AD)-related degenerative decline is associated to the presence of amyloid beta (Aβ) plaque lesions and neuro fibrillary tangles (NFT). However, the precise molecular mechanisms linking Aβ deposition and neurological decline are still unclear. Here we combine genome-wide transcriptional profiling of the insular cortex of 3xTg-AD mice and control littermates from early through to late adulthood (2–14 months of age), with behavioral and biochemical profiling in the same animals to identify transcriptional determinants of functional decline specifically associated to build-up of Aβ deposits. Differential expression analysis revealed differentially expressed genes (DEGs) in the cortex long before observed onset of behavioral symptoms in this model. Using behavioral and biochemical data derived from the same mice and samples, we found that down but not up-regulated DEGs show a stronger average association with learning performance than random background genes in control not seen in AD mice. Conversely, these same genes were found to have a stronger association with Aβ deposition than background genes in AD but not in control mice, thereby identifying these genes as potential intermediaries between abnormal Aβ/NFT deposition and functional decline. Using a complementary approach, gene ontology analysis revealed a highly significant enrichment of learning and memory, associative, memory, and cognitive functions only among down-regulated, but not up-regulated, DEGs. Our results demonstrate wider transcriptional changes triggered by the abnormal deposition of Aβ/NFT occurring well before behavioral decline and identify a distinct set of genes specifically associated to abnormal Aβ protein deposition and cognitive decline.

## Introduction

Alzheimer’s disease (AD) is a degenerative disease characterized by progressive memory impairment and loss of other cognitive functions for which there is no current known effective treatment. In 2010, 46.8 million people worldwide were diagnosed with dementia and the number of cases is expected to reach almost 74.7 million cases by 2030 (Prince, 2015), with AD accounting for over 70% of all reported cases (Ferri et al., 2005). While AD is more common in people over 65 years of age, with age being the most prominent risk factor, several additional risk factors have been identified including brain injury, smoking, and even low social engagement (Hersi et al., 2017). However, their predictive value is limited.

AD is characterized by the progressive loss of synaptic density and synapse number followed by overt neuronal degeneration involving the breakdown of neuronal maintenance and survival mechanisms (Selkoe, 2001). Key neuropathological correlates of AD are the presence of distinctive lesions comprised of diffuse and neuritic plaques predominantly composed of; (i) extracellular plaques of aggregated amyloid-beta (Aβ) protein which have been linked to neurotoxicity and axon pruning (Bharadwaj et al., 2009; Nikolaev et al., 2009), and (ii) intraneuronal tangles formed by aggregates of hyper-phosphorylated neurofilament-associated protein tau -known as tau neurofibrillary tangles (NFT)- (Selkoe, 2001; Querfurth and LaFerla, 2010) reported to affect microtubule stability, altering the cell’s cytoskeleton and collapses the neuron’s transport system (Iqbal et al., 2005). Histological evidence indicates that Aβ accumulation precedes and drives tau aggregation (Lewis et al., 2001; Oddo et al., 2003b). Abnormal accumulation of Aβ is the result of an imbalance between the levels of Aβ production, aggregation and clearance (Wang et al., 2010). Aβ clearance is mediated by proteolytic enzymes like neprilysin (Iwata et al., 2001), chaperone molecules such as apoE (Kim et al., 2009), lysosomal (Bendiske and Bahr, 2003), and non-lysosomal pathways (Marambaud et al., 2005). However, the precise identity of the wider molecular pathways that become compromised during the gradual build-up of abnormal Aβ deposition and NFT remain largely unknown (Selkoe and Hardy, 2016).

The use of animal models of AD can help overcome some of the ethical and technical limitations of studying human patients. While acknowledging the intrinsic limitations associated to any preclinical model, animal models of AD offer a unique opportunity to understand the progression of this condition as samples can be extracted from individuals at different ages including those not yet symptomatic and in controlled circumstances. Importantly, in addition to the experimental advantage of a model with a much shorter lifespan, life events can be kept largely homogeneous across individuals in a given study. Currently more than 50 different animal models have been generated to explore AD (Stover et al., 2015). Amongst the AD mouse models currently available, the 3xTg-AD model reported by Oddo et al. (2003a,b) is one of the models that show higher similarity to the brain pathology of AD observed in humans. This model is also one of the few models that has been shown to develop both Aβ plaques and NFT as well as well-known neurochemical hallmarks such as gradual dysfunction of the cholinergic and dopaminergic systems; both believed to contribute to the age-related cognitive decline in AD (Guzmán-Ramos et al., 2012b; Do et al., 2014; Moreno-Castilla et al., 2016; Selkoe and Hardy, 2016). The 3xTg-AD mouse model has two different polymorphisms associated with AD in humans: presenilin-1 (PSEN1), Aβ precursor protein (APP) as well as a mutant allele of the microtubule-associated protein tau (MAPT). These mice are viable, fertile and display no initial gross physical abnormalities, while presenting abnormal Aβ deposition and NFTs, with early brain lesions being observable as early as 6th month of age (Oddo et al., 2003b; Caruso et al., 2013). The 3xTg-AD mice have been shown to display learning and memory deficits in a wide range of learning and memory tests including Barnes maze, Morris water maze (Oddo et al., 2006), object recognition memory, conditioned taste aversion (CTA) and others (Chin, 2011; Guzmán-Ramos et al., 2012b; Moreno-Castilla et al., 2016).

Here we use the CTA learning paradigm as a simple, robust and experimentally tractable learning model known to be mediated by a well-defined cortical region (the insular cortex, Guzmán-Ramos et al., 2012a; Moreno-Castilla et al., 2016; Wu et al., 2020), to detect molecular mediators linking the progressive build-up of Aβ aggregates with cognitive decline. More specifically, we combine genome-wide transcriptional profiling of the cortex of 3xTg-AD mice and control littermates before, during and after the onset of behavioral symptoms (2–14 months of age) with behavioral and biochemical profiling in the exact same mice, to identify wider transcriptional determinants of functional decline specifically associated to the progressive build-up of Aβ deposition. Our results demonstrate wider transcriptional changes occurring well before behavioral decline triggered by the abnormal deposition of Aβ taking place in this model, and identify a distinct set of genes specifically associated to both Aβ protein build up and behavioral decline.

## Materials and Methods

### Behavioral Assessment

Mice crosses to obtain 3xTg-AD homozygous (from the Jackson Laboratory) and corresponding controls were performed as described elsewhere (Guzmán-Ramos et al., 2012a). Only male mice were used to maintain homogenous conditions and avoid hormonal-driven behavioral variations affecting the learning task readouts. Prior to tissue sample collection, animals were tested for learning and memory performance at 2, 7, 8, 11, and 14 months of age using conditioned taste aversion (CTA) assay as described by Moreno-Castilla et al. (2016). This behavioral test consists of pairing a novel flavor (a single presentation of a saccharin solution) with a gastric malaise stimulus induced via an IP injection of lithium chloride (LiCl). After 24 h from the initial presentation of saccharine solution and LiCl injection, aversion to the novel flavor was assessed by measuring the level of consumption of a saccharin solution compared to water. An aversion measure was calculated dividing the saccharin solution intake by the sum of the baseline water consumption and saccharin solution intake.

### Tissue Sample Collection

After CTA memory testing, mice were euthanized by cervical dislocation. Insular cortex was bilaterally dissected and tissue from both hemispheres was aggregated into a single sample per animal. Samples were taken from a total of 30 male mice, three wild-type mice and three 3xTg-AD mutant mice at each chosen postnatal age. Tissue samples were stored in RNA-Later^R^ at −80C until further analysis.

### Expression Data

PolyA-selected *Illumina RNA-seq* was performed on one hemisphere of the insular cortex dissected from each mouse. The *FastQC* tool was used to evaluate the quality of the data. *Trimmomatic* software was employed to filter poor quality reads and remove Illumina adapters. Transcriptome annotation was carried out using *tophat2, samtools* and *R* software (*version 3.2.2 (2015-08-14)—“Fire Safety”*). Genes that displayed zero variance across samples and those that had a cumulative RPKM value of less than five across all samples were discarded, leaving total of 16,894 protein coding genes. The RPKM expression data, together with behavioral and Aβ-42 deposition data is available in the GEO public database (accession number: GSE161904).

### Differential Expression Analysis

Differential expression analyses were carried out with the edgeR package supported in R (Robinson et al., 2010) using TMM normalization as proposed by Robinson and Oshlack (2010) and the exact test model (Robinson and Smyth, 2008). Differentially expressed genes (DEGs) were identified by comparing wild type and 3xTg-AD samples for each time point separately.

### Functional Enrichment Analysis

Enrichment analyses of functional annotations of Gene Ontology (GO) terms was performed after pooling up and down-regulated DEGs across time points in the 3xTg-AD mouse model after pooling. We tested for significant over-representation of specific gene ontologies using the WEB-based Gene SeT AnaLysis Toolkit (WebGestalt) available online (Zhang et al., 2005). Overrepresented functional terms were identified after correcting for multiple testing.

### Aβ Deposition Quantification

Amyloid Betta (isoforms 42 and 41) deposition was quantified using the milliplex enzyme-linked immunosorbent assay (ELISA) platform. Quantification assays were performed twice (2 × *n* = 3 animals per group of age) following manufacturer’s guidelines with duplicate measurements per sample. We used a highly sensitive milliplex^®^ MAP Kit (Cat # HNABTMAG-68K, Millipore) with color-coded microspheres (beads) and fluorescent dyes; through precise concentrations, the beads simultaneously and specifically capture amyloid beta 1–40 and 1–42. A total of 25 μL of sample was introduced into a plate containing the microspheres. After an analyte from a test sample is captured by the bead, a biotinylated detection antibody is introduced. The reaction mixture was then incubated with Streptavidin-PE conjugate, the reporter molecule, to complete the reaction on the surface of each microsphere. To acquire and analyze data, we used a Luminex^®^ MAGPIX^®^ instrument where each individual microsphere was identified and the result of its bioassay was quantified.

### Randomization Test

To assess the possible relationship between the DEGs in the cortex with Aβ deposition and learning performance of each mouse, we obtained absolute Pearson correlation coefficients for the expression levels for each gene in all the mice across all five developmental stages against both Aβ deposition and aversion index. A randomization test was carried out to assess the significance of the association between gene expression patterns with Aβ deposition and aversion index for DEG genes. For this, we calculated the average of the absolute correlation coefficients between gene expression levels and either Aβ deposition or the aversion index for all DEG genes. This mean value was then compared to the mean value of absolute correlation coefficients of 1,000 equally sized randomly selected samples of background genes. Association between the expression of DEGs and either Aβ deposition or behavioral performance was regarded as significant if the mean absolute correlation for DEGs was greater than the largest mean correlation value observed in the random background gene samples (*p* < 0.001). All large-scale calculations were carried out using the programming language R and associated libraries.

## Results

**FIGURE 1:**
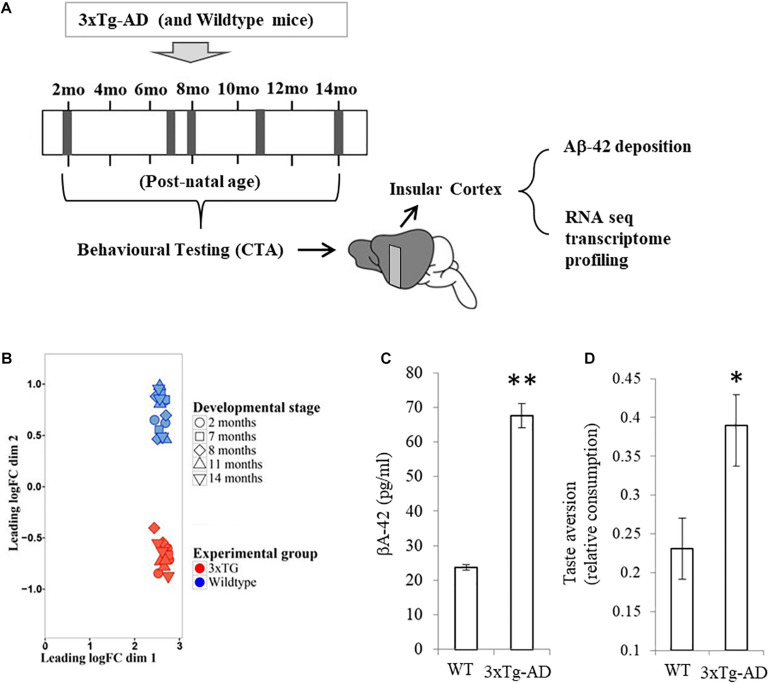
Experimental groups and expression profile clustering. **(A)** Samples were collected from cerebral cortex of three wild type mice and three 3xTg-AD mutants at different time points throughout adult development (2, 7, 8, 11, and 14 months of age, indicated by gray vertical bands in the timeline). At the end a total of 30 samples were obtained. **(B)** Multidimensional scaling (MDS) plot of the TMM normalized data. blue symbols represent wild type mice and red symbols were used to denote 3xTG-AD mice. **(C)** BA-42 concentration in the same samples (± SEM) for both wild type (WT) and 3xTg-AD mice. **(D)** Learning performance in the conditioned taste aversion task in the same mice. Each bar represents mean relative consumption (± SEM) of the aversively associated flavor relative to mean base-line consumption of water. ^∗^*p* < 0.05, ^∗∗^*p* < 10^–10^.

In order to characterize the transcriptional changes that occur in the insular cortex in response to the build-up of Aβ/NF deposits and cognitive deterioration in the 3xTg-AD mouse model, behavioral data and tissue samples were obtained from three transgenic and three wild-type male mice at five time points from two through to 14 months of age (*n* = 3/time point, Figure 1A). Cognitive performance assessment was carried out using the conditioned taste aversion (CTA) learning task (see section “Materials and Methods”). Learned acquisition of aversion for a novel flavor is known to be mediated by the insular cortex and found to be severely impaired in adult 3xTg-AD mice when compared with normal mice (Guzmán-Ramos et al., 2012a; Moreno-Castilla et al., 2016). The time points chosen were based on the fact that cognitive impairment in 3xTg-AD mice has been documented in some memory tests as early as 4 months (Stover et al., 2015). Accordingly we have based our choice on the time course previously characterized by Moreno-Castilla et al. (2016), where learning deficits in Conditioned taste aversion were observed only after the 8th moth of age (Guzmán-Ramos et al., 2012b; Moreno-Castilla et al., 2016). All animals were euthanized immediately after the CTA memory testing trial at each chosen age and the insular cortex was bilaterally dissected for Aβ quantification and transcriptional profiling.

Transcriptional profiling on tissue samples was carried out using the Illumina RNA-seq platform for all 3xTg-AD and wild type mice for each time point. After transcriptome annotation and removal of genes with less than five reads overall and those with no variance, gene expression levels were compiled for a total of 16,894 protein-coding genes. TMM normalization was applied to make sure expression among samples is comparable. Multidimensional scale analysis revealed two distinct clusters separating each experimental group (Figure 1B). No sub-clustering was observed by age. This result demonstrates that significant gene expression differences are apparent in all time-points between 3xTg-AD and WT samples. Quantification of beta amyloid deposition (isoform Aβ-42) measured on the exact same samples show a statistically significant difference between 3xTg-AD and WT samples (Figure 1C, *t* = 11.9, *p* = 4.7 × 10^–12^). Taste aversion learning performance carried out on the same mice prior to tissue sampling also reveals a statistically significant difference in consumption of novel flavor relative to base-line water consumption (Figure 1D, *t* = 2.4, *p* = 0.023).

**FIGURE 2:**
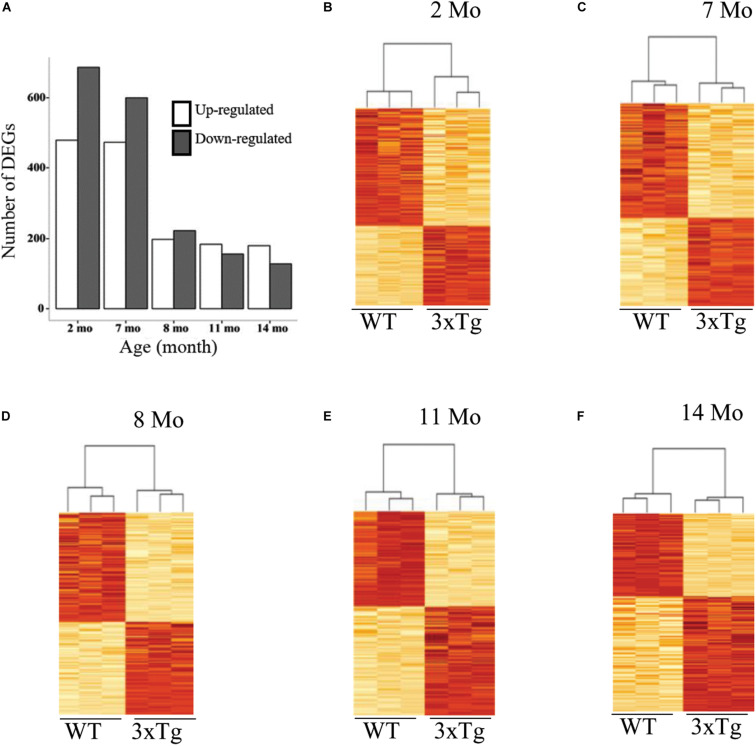
Time course of differential gene expression in 3xTg-AD mice. **(A)** Bar charts showing the number of differentially expressed genes (DEGs) in the cortex both up and down-regulated, on the vertical axis, as a function of age in months (horizontal axis). **(B–F)** Heatmaps and hierarchical clustering of triplicate samples corresponding to each of the five time points studied (indicated in months). Each plot displays, on the vertical axis, the pulled set of DEGs detected across all five ages (*n* = 1924), with the highest color intensity corresponding to the highest expression (z score-transformed).

To assess gene expression differences, in the insular cortex, between 3xTg-AD and WT we carried out a differential expression analysis at each time point. This analysis revealed a distinctly decreasing temporal trend in the number of genes differentially expressed in 3xTg-AD samples relative to WT samples between 2 and 14 months of age in the insular cortex, with the most drastic decrease in the number of differentially expressed genes (DEGs) between 7 and 8 months. This pattern was consistent for both up-regulated and down-regulated genes (Figure 2). This temporal trend contrasts with the known symptomatic manifestation of cognitive decline in the 3xTg-AD mouse model as behavioral decline increases as individuals become older (Guzmán-Ramos et al., 2012b; Stover et al., 2015; Moreno-Castilla et al., 2016). This result suggests that most transcriptional alterations take place at early stages long before behavioral decline become apparent in this model as measured by CTA learning.

**FIGURE 3:**
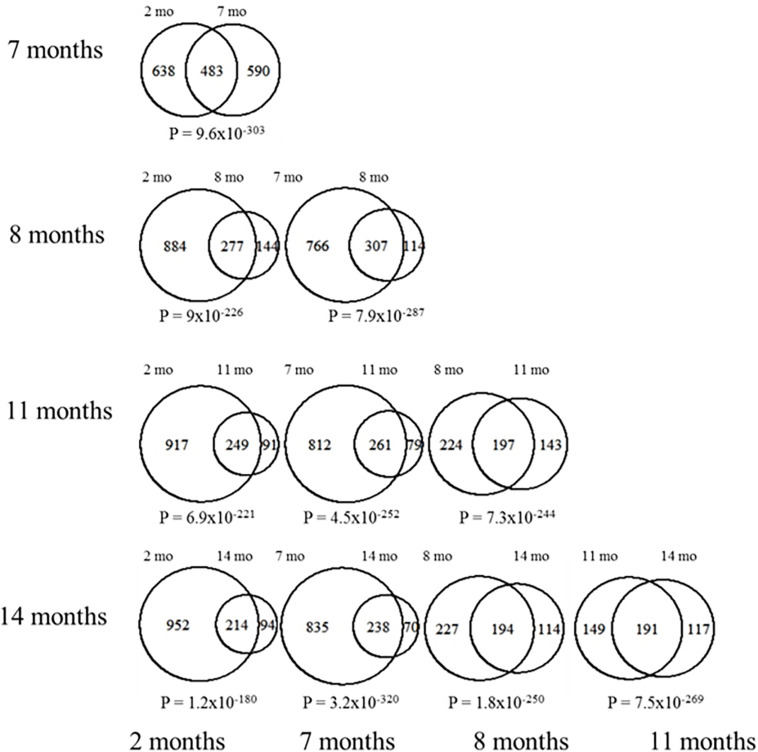
Pairwise DEG overlap between all different ages. Each Venn diagram shown represents the overlap in DEGs between the indicated ages. The numbers of DEGs at each stage are also indicated. Chi square tests were used to assess the significance in the observed overlap relative to chance expectations, the corresponding *p*-values are indicated under each of the Venn diagrams.

We then assessed the proportion of overlapping DEGs across all time points to determine if there was a clearly distinct set of differentially expressed genes at early or late stages of neurological decline progression. Pairwise comparisons between DEGs at each time point reveal that there is a highly statistically significant proportion of overlapping genes across all age comparisons (Figure 3). This result confirms the existence of a common core of differential expression signature associated to the cortical deposition of Aβ in the 3xTg-AD model. In all the subsequent analyses we use the complete pool of all differentially expressed genes across all developmental stages (*n* = 1,924), including up (*n* = 829) and down (*n* = 1,125) regulated genes separately (listed in Supplementary Table 1).

**FIGURE 4:**
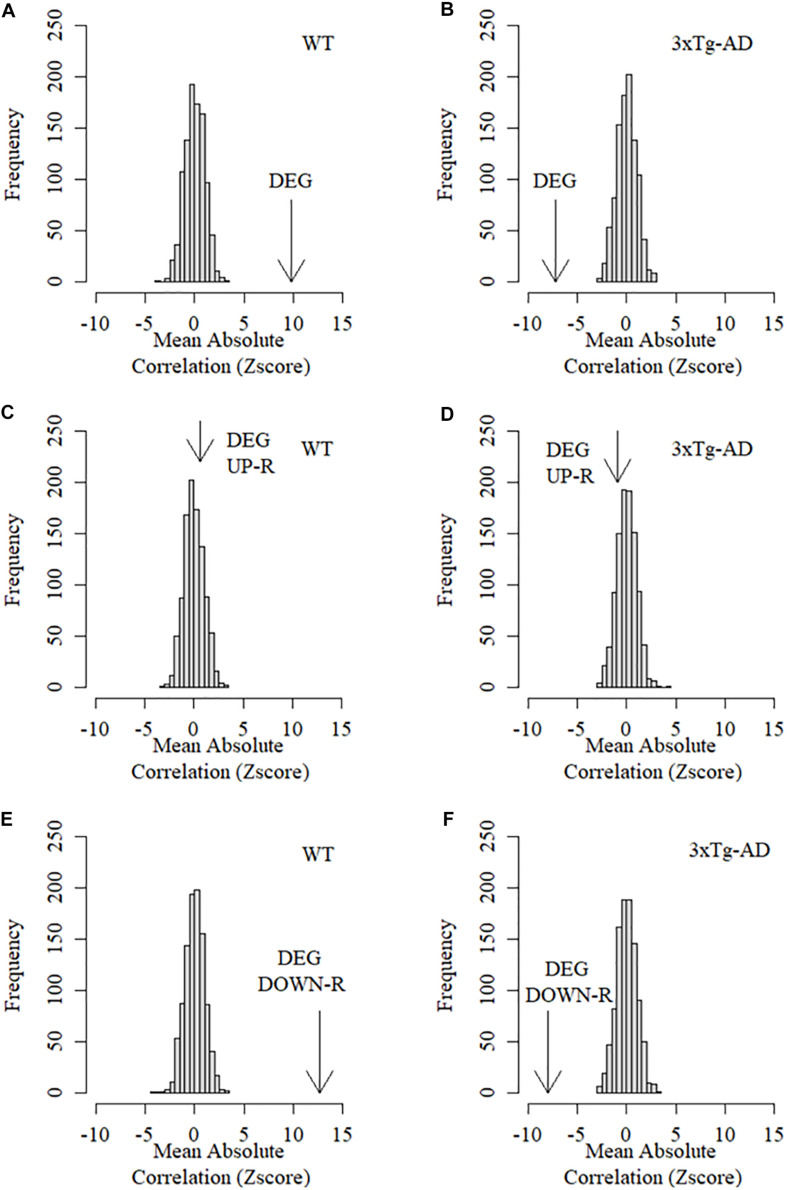
Mean association between behavioral performance and expression level of DEGs in the cortex of control and 3xTg-AD mice. Arrows in these graphs represent the Z score of the mean absolute correlation between expression of DEGs and CTA behavioral performance relative to the correlation distribution (z score-transformed) of 1,000 equally sized samples of random background genes. **(A)** Mean association between differentially expressed genes and learning performance in the cortex of WT mice. **(B)** Mean association between differentially expressed genes and learning performance in the cortex of 3xTg-AD mice. **(C)** Mean association between up-regulated differentially expressed genes and learning performance in the cortex of WT mice. **(D)** Mean association between Up-regulated differentially expressed genes and learning performance in the cortex of 3xTg-AD mice. **(E)** Mean association between down-regulated differentially expressed genes and learning performance in the cortex of WT mice. **(F)** Mean association between Down-regulated differentially expressed genes and learning performance in the cortex of 3xTg-AD mice.

While the large number of DEGs observed suggest the existence of widespread alterations in expression patterns in 3xTg-AD mice, many of these changes may not be directly linked to the mechanisms of CTA learning decline in AD as they could be the result of spurious collateral effects on gene expression. In order to assess whether DEGs are specifically associated to learning decline, we used learning performance data based on the CTA test obtained from the exact same mice prior to transcriptome profiling and calculated the absolute Pearson correlation coefficients between the level of expression for each gene against the acquired taste aversion index across all replicas and time points. Absolute correlation coefficients were next calculated separately for control and 3xTg-AD mice. The mean absolute correlation coefficient for DEGs was then compared to mean absolute correlations resulting from a thousand equally sized random samples of background genes. As shown in Figure 4A, the collective association between DEGs with CTA learning performance was found to be significantly greater than expected by chance in WT cortex, demonstrating that DEGs are linked to cortically-mediated learning performance in normal mice. This relationship was completely disrupted in the cortex of 3xTg-AD mice where the mean association between expression and CTA learning performance of the same DEGs was actually less than that observed in random gene sets (Figure 4B). This finding strongly suggests an actual functional link between AD pathogenesis and DEGs. However, it is unlikely that all of them participate in the mechanistic onset of AD-related functional decline as many of them could be the result of spurious collateral regulatory alterations not directly linked with this condition. We therefore examined up and down-regulated genes separately in order to assess their relative association with CTA learning. Down-regulated DEGs showed a much more significant association with CTA learning in WT mice than the whole set of DEGs (Figure 4C). This association was again dramatically disrupted in 3xTg-AD mice (Figure 4D). By contrast, the collective association of up-regulated DEGs with CTA learning was no different than that of random background genes in both WT and 3xTg-AD mice (Figures 4E,F). Taken together, these results show that only down-regulated differentially expressed genes show a stronger average association with insular cortex-mediated learning performance than random background genes in control but not 3xTg-AD mice.

**FIGURE 5:**
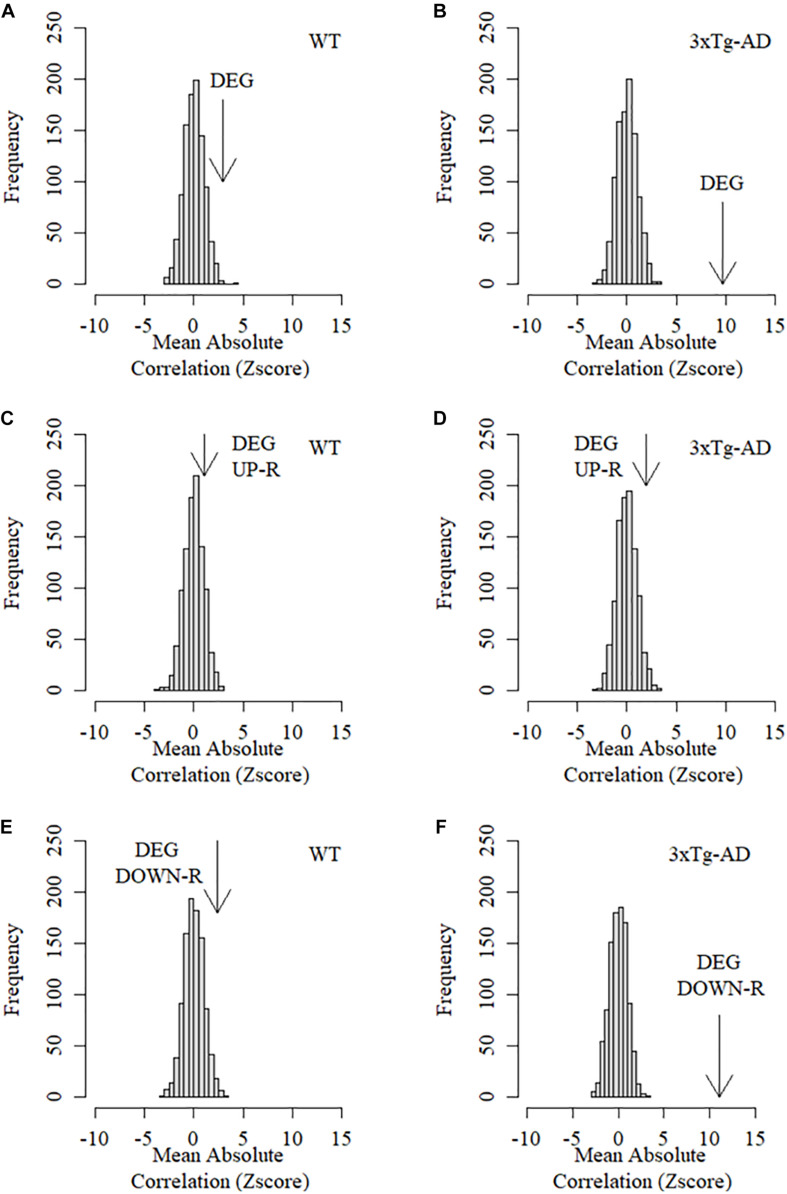
Mean association between Aβ-42 protein deposition and expression level of DEGs in the cortex of control and 3xTg-AD mice. Arrows in these graphs represent the Z score of the mean absolute correlation Between expression of DEGs and Ba42 deposition relative to the correlation distribution (z score transformed) of 1,000 equally sized samples of random background genes. **(A)** Mean association between differentially expressed genes and Aβ-42 deposition in the cortex of WT mice. **(B)** Mean association between differentially expressed genes and Aβ-42 deposition in the cortex of 3xTg-AD mice. **(C)** Mean association between up-regulated differentially expressed genes and Aβ-42 deposition in the cortex of WT mice. **(D)** Mean association between Up-regulated differentially expressed genes and Aβ-42 deposition in the cortex of 3xTg-AD mice. **(E)** Mean association between down-regulated differentially expressed genes and Aβ-42 deposition in the cortex of WT mice. **(F)** Mean association between Down-regulated differentially expressed genes and Aβ-42 deposition in the cortex of 3xTg-AD mice.

We then assessed whether the expression patterns of DEGs (up and down-regulated) were specifically associated with amyloid beta protein deposition in the insular cortex. To this end, we assessed the collective association between DEGs and Aβ by calculating the mean absolute Pearson correlation coefficient between the level of expression for each and all DEGs across all replicas and time points, and the deposition of Aβ (isoform 42) measured in the exact same samples (see section “Materials and Methods”). We next compared the mean absolute correlation between expression of DEGs and Aβ deposition with the mean correlations resulting from a thousand equally sized random samples of background genes. This analysis revealed that cortical DEGs displayed significantly higher mean absolute correlation with Aβ deposition only in 3xTg-AD but not WT cortical samples (Figure 5A). Neither up nor down-regulated DEGs showed a significant association with Aβ42 in WT mice (Figures 5B,D,F). However, in 3xTg-AD mice, only down-regulated DEGs displayed a highly significant association with Aβ-42 deposition with up-regulated genes being no different to random background genes (Figures 5C–E). Taken together, these results demonstrate that normal expression of down but not up-regulated DEGs is highly associated with CTA learning performance and that this association is drastically disrupted in 3xTg-AD mice. Conversely, these same down-regulated DEGs are strongly associated with cortical Aβ deposition in 3xTg-AD mice but not WT mice strongly indicating that this set of genes is directly involved in the molecular events linking Aβ deposition and AD-related functional decline.

**FIGURE 6:**
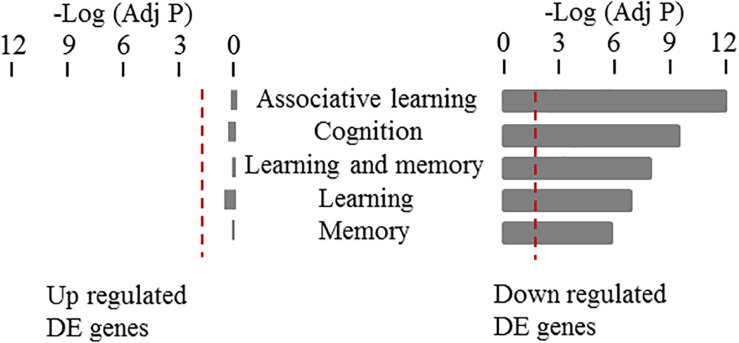
Gene ontology enrichment analysis of differentially expressed genes in the cortex of 3xTG-AD mice. Enrichments on up- and down-regulated DEGs in cortex are shown for genes annotated to the following functional categories associated to learning and memory mechanisms: Learning or Memory (GO:0007611), learning (GO:0007612), memory (GO:0007613), cognition (GO:0050890), associative learning (GO:0008306). Bars show the probability values after Benjamini-Hochberg correction for multiple testing in a negative logarithm scale for each of the functional categories tested. Only cortical down-regulated genes showed a statistically significant enrichment with these categories.

Using a complementary approach, we used gene ontology enrichment analysis to test the association between learning and memory related specific cellular functions and our identified set of differentially expressed genes in 3xTg-AD mice. Specifically, we looked for a potential enrichment of specific functional categories among the up- and down-regulated genes using the WEB-based Gene SeT AnaLysis Toolkit (WebGestalt) (Zhang et al., 2005). We looked separately at the sum of all the up- and down-regulated DEGs across all five time points analyzed. Then, Gene Ontology Analysis (GO Analysis) was conducted in each of the four sets of genes; namely: up- and down-regulated DEGs (listed in Supplementary Table 1). We specifically examined any potential over representation of functional categories generally associated to learning and memory mechanisms (rather than mechanisms specific for taste aversion learning) including: Learning or Memory (GO:0007611), learning (GO:0007612), memory (GO:0007613), cognition (GO:0050890), associative learning (GO:0008306). As shown in Figure 6, down but not up-regulated DEGs showed a highly significant enrichment of these functional categories (a complete analysis of GO term enrichment at each time point for either up or down regulated DEGs is shown in Supplementary Table 2). Taken together these results confirm the specific association of down but not up-regulated DEGs in CTA learning and memory functions and support their involvement in linking progressive Aβ deposition in AD and learning performance deterioration.

## Discussion

In the present study, we conducted the RNA-seq gene expression profiling of the insular cortex of 3xTg-AD mouse model and performed a differential expression analysis to identify the changes in gene expression that occur throughout adulthood (2–14 months), both at symptomatic and pre symptomatic stages, in this animal model. Animal models of AD have become a widely accepted alternative to emulate human AD and offer the opportunity to track molecular and functional alterations associated to AD at different stages of progression of this disease.

Differential expression analysis comparing 3xTg-AD mice with wild-type mice revealed that, in the insular cortex, the largest proportion of gene expression changes occur at very early adult stages (2–7 months) and way before the known onset of learning decline in this model and that the actual number of differentially expressed genes decreases as these mice become older. Interestingly, 8 month of age seems to be a critical stage for the 3xTg-AD model as it is at this time point where the number of DEGs decreases drastically in the insular cortex and remains around the same during the following stages. This temporal trend contrasts with the known symptomatic manifestation of cognitive decline in the 3xTg-AD mouse model as cognitive decline (as measured by CTA learning) increases as individuals become older (Guzmán-Ramos et al., 2012b; Stover et al., 2015; Moreno-Castilla et al., 2016). While cognitive impairment in 3xTg-AD mice has been as documented in some memory tests as early as 4 months (Stover et al., 2015), learning deficits in conditioned taste aversion are typically observed only after the 8th month of age (Moreno-Castilla et al., 2016), Aβ plaques and LTP/LTD alterations are detected at around 6 months (Oddo et al., 2003a,Oddo et al., 2003b), with gliosis becoming fairly evident at 7 months of age (Caruso et al., 2013). The formation of NFTs, however, is not evident until 12 months of age (Oddo et al., 2006), and it has been suggested that NFTs could contribute to the vascular volume abnormalities observed in the 3xTg-AD mouse model, which appear as early at 6 months of age and are consistently present up until 18 months of age (Do et al., 2014). For the behavioral test used in this study (Conditioned taste aversion), our findings suggest that, during the normal progression of this condition, a large number of critical gene regulatory alterations are likely to take place at rather early adult stages and long before any learning symptoms become apparent. This in turn opens the tantalizing possibility of identifying central (and potentially peripheral) biomarkers capable of predicting future development of this condition at much earlier stages.

Our finding that, in this model, the number of differentially expressed genes follow a decreasing trend in time, as the actual pathology aggravates, also suggests that most gene expression changes measured in post-mortem samples obtained from AD patients could represent a small proportion of the most critical gene expression alterations effectively linked to the initial onset of this condition, a circumstance that could also hold for other neurological conditions (i.e., Parkinson’s disease, schizophrenia, and others). In addition, it is worth noting that many of these changes could actually follow opposing trends at different time points, and at early asymptomatic stages. Thus for instance, dopaminergic functions have been studied in the context of AD-related pathology (Kar et al., 2004; Guzmán-Ramos et al., 2012a; Puzzo and Arancio, 2013; Moreno-Castilla et al., 2016), with key alterations including reduced secretion of dopamine, reduced expression of the dopamine receptor (mainly D2 subtypes) and lower dopamine active transporter (DAT) expression in the hippocampus and frontal cortex (Alkondon and Albuquerque, 2002; Palop and Mucke, 2010), along with reduced dopamine transmission through D1 and D2 receptors (Iqbal et al., 2013). Among the differentially expressed genes we identify in this study, is precisely the dopamine receptor D2. While this receptor appears normally expressed at late stages (11th an 14th months), it was found significantly up-regulated during the second month to then become significantly down-regulated by the 8 month of age, suggesting a link between these early gene regulatory disturbances and subsequent dopaminergic dysfunctions observed in AD patients.

In this study, we combined temporal RNA-seq transcriptional profiling of the brain cortex in 3xTg-AD mice with behavioral and biochemical readouts obtained from the exact same animals (i.e., learning performance in the CTA test and Aβ-42 deposition measured in the same animals and tissue samples). As far as we know, a combined approach of this nature in AD models has never been attempted before. By looking at correlations between the time-course of gene expression and either, CTA learning and memory performance on the one hand, or Aβ deposition on the other, we are able to identify transcriptional determinants specifically associated with both amyloid deposition and progressive functional decline. While conventional differential expression profiling can identify scores of genes abnormally regulated in this condition, a large proportion of them are likely to be the result of spurious collateral regulatory alterations not directly linked to underlying mechanisms of disease progression. Indeed, we found that among all differentially expressed genes, only down-regulated DEGs were, collectively, strongly associated with CTA learning in control mice when compared with random background genes, as shown by our randomization analysis. This strong collective association was found severely disrupted in the 3xTg-AD mice. Conversely, these same genes were found to be strongly associated with Aβ deposition only in 3xTg-AD mice, but not controls. On the other hand, the subset of up-regulated DEGs were no more associated to learning performance or Aβ deposition than random background genes in both control and 3xTg-AD mice. The fact that a very specific set of genes undergoes a distinct switch from displaying a strong association with CTA learning in healthy mice to a strong association with gradual Aβ deposition in 3xTg-AD mice strongly points toward these specific genes as key mediators of the cognitive decline triggered by the gradual Aβ imbalances taking place during AD progression.

Using a complementary approach based on gene ontology enrichment analysis, we further tested the potential overrepresentation of biological functions associated with learning and memory mechanisms among either up or down-regulated DEGs. This analysis confirmed a specific and highly significant enrichment of learning and memory-related biological processes exclusively among down, but not up-regulated DEGs.

Taken together, these results identify, by combining behavioral and biochemical data with transcriptional profiling, a subset of differentially expressed genes specifically associated with progressive beta amyloid deposition on the one hand and CTA learning decline on the other.

Few previous studies have looked into overall patterns of differential expression in mouse models of AD. And usually these have been carried out at a single time point (Reddy et al., 2004; Mirnics et al., 2005; Unger et al., 2005; Kim k. H. et al., 2012; Kim T.-K. et al., 2012; Castillo et al., 2017). However, changes in the time course of expression patterns before, during and after the onset of functional decline specifically linking Aβ/NFT imbalances with learning decline remain poorly understood, with only two studies recently reporting data on general temporal changes in expression patterns in two mouse models. One study was carried out in 3xTg-AD mice using comparative microarray profiling of hippocampal expression patterns at 3 and 12 months using an experimental set up aimed at identifying common genetic mechanisms shared by aging and AD-like backgrounds (Gatta et al., 2014). While the cited study identifies a number of up and down-regulated hippocampal genes shared by young 3xTg-AD and normal aging mice, (suggesting the early onset of aging-related mechanisms during AD progression), it remains uncertain which of the observed changes are directly responsible for the link between Aβ/NFT imbalances and functional decline as opposed to collateral transcriptional changes not directly involved in AD progression. A second study recently reported was carried out on the 5XFAD mouse model (a model carrying five mutations associated to the familial form of AD) at four time points, two before and two after the onset of cognitive decline in this model (Landel et al., 2014). While, compared with other models, 5XFAD mice display AD features much earlier but with no clear tau pathology, a consistent signature of differential expression reveals that many of the DEGs specific to the 5XFAD model belong to neuroinflammatory processes typically associated with plaques (Bouter et al., 2014; Landel et al., 2014). It is worth noting that the time course of differential expression in 5XFAD mice shows an increasing rather than decreasing trend in the number of DEGs as time progresses (Landel et al., 2014). This increasing trend in the number of DEGs starkly contrasts with the decreasing trend we found in the cortex of 3xTg-AD mice where the largest number of DEGs was observed at 2 months of age with a gradual reduction at later stages after the onset of functional decline in these mice. This contrast between the two models suggests differences in the time course of gene regulatory alterations triggered by the different transgenic backgrounds. Our finding that only a subset of differentially expressed genes display a direct association with both amyloid deposition and changes in learning performance illustrate that a large proportion of differential expression events are likely to be the result of spurious collateral regulatory alterations not directly linked with the underlying mechanisms of AD pathogenesis. Future studies aligning transcriptional profiling with changes in behavioral and biochemical parameters in 5XFAD mice, will help gain critical insights into the molecular mechanisms specifically associated to disease development in this preclinical model.

While current animal models offer an invaluable opportunity to gain critical insights into such a complex pathology as AD and neurodegeneration in general, caution is necessarily needed when extrapolating to the human context, and further work will be needed to properly bridge the gap between our current understanding of this condition, based on animal models, and the clinical settings.

In summary, our analyses of the time course of transcriptional alterations in the 3xTg-AD mice combined with biochemical and behavioral profiling in the same mice, reveal widespread changes in gene expression, most of which taking place long before the onset of functional decline in this model (as assessed using the CTA learning paradigm), opening the possibility of identifying key biomarkers at very early pre-symptomatic stages of the disease. We additionally found that only down-regulated DEGs show a specific collective association with CTA learning performance in healthy mice and that this association is severely disrupted in 3xTg-AD mice. Conversely, this same subset of DEGs displays a specific, collective, association with gradual increases in Aβ deposition in 3xTg-AD mice but not in healthy mice, suggesting that these genes constitute a central part of the machinery that directly links gradual imbalances in Aβ clearance on the one hand and cognitive decline in AD.

## Data Availability Statement

The original contributions presented in the study are publicly available. This data can be found here: NCBI GEO: GSE161904.

## Ethics Statement

The animal study was reviewed and approved by the Universidad Nacional Autonoma de Mexico.

## Author Contributions

AC-M, PM-C, FB-R, AOU, and HG conceived and designed the study. AC-M, JM-S, PM-C, and RP-O collected the tissue samples and carried out the behavioral testing. PM-C and RP-O carried out the biochemical analysis of brain samples. MLR-T-S, NC-H, and AC-M annotated transcriptome data and carried out differential gene expression analyses. WY and NC-H conducted the correlation analyses on transcriptional, behavioral, and biochemical data. NC-H, MLR-T-S, AOU, and HG wrote the manuscript. All authors read and approved the final version.

## Conflict of Interest

The authors declare that the research was conducted in the absence of any commercial or financial relationships that could be construed as a potential conflict of interest.

## Abbreviations

A β: Beta Amyloid protein
APP: Amyloid precursor protein
AD: Alzheimer’s Disease
CTA: Conditioned Taste Aversion test
DAT: Dopamine active transporter
DEG: Differentially expressed genes
GO: Gene ontology
LTP/LTD: Long term potentiation/depression
NFT: Neurofibrillary tangles
MAPT: microtubule-associated protein tau
3xTg-AD: Triple transgenic AD mouse model.
