# Supplementary material for: Transcriptional, Behavioral and Biochemical Profiling in the 3xTg-AD Mouse Model Reveals a Specific Signature of Amyloid Deposition and Functional Decline in Alzheimer’s Disease
Source: Front Neurosci. 2020 Dec 15;14:602642. doi: 10.3389/fnins.2020.602642 (PMC7774037; doi:10.3389/fnins.2020.602642)
Supplement: Supplementary file 2 [file Data_Sheet_2.PDF]

Supplementary Table 2
Gene ontology enrichment analysis for genes differentially expressed in 3xTg-AD mice relative to Controls, detected at each individual time point included in the study.

Biological Process Enrichment: 2 months (Up-regulated DEGs)

GO ID:	Description	FDR (Benjamini-Hochberg)
GO:0007610	behavior	8.42E-06
GO:0001975	response to amphetamine	3.32E-05
GO:0014075	response to amine	7.58E-04
GO:0043279	response to alkaloid	8.94E-04
GO:1904062	regulation of cation transmembrane transport	8.94E-04
GO:0097366	response to bronchodilator	9.70E-04
GO:0043270	positive regulation of ion transport	9.70E-04
GO:0003008	system process	9.70E-04
GO:0007626	locomotory behavior	0.001222
GO:0007631	feeding behavior	0.001222
GO:0010959	regulation of metal ion transport	0.001222
GO:0008306	associative learning	0.002448
GO:0034765	regulation of ion transmembrane transport	0.002532
GO:0072347	response to anesthetic	0.002687
GO:0007612	learning	0.002687
GO:0007611	learning or memory	0.002687
GO:0035815	positive regulation of renal sodium excretion	0.003102
GO:0034767	positive regulation of ion transmembrane transport	0.00342
GO:0043269	regulation of ion transport	0.003495
GO:2001025	positive regulation of response to drug	0.003747
GO:0035094	response to nicotine	0.004033
GO:0050801	ion homeostasis	0.004091
GO:0042220	response to cocaine	0.004091
GO:0051050	positive regulation of transport	0.004091
GO:1904064	positive regulation of cation transmembrane transport	0.00414
GO:0098801	regulation of renal system process	0.004158
GO:0098655	cation transmembrane transport	0.005991
GO:0009636	response to toxic substance	0.005991
GO:0050890	cognition	0.005991
GO:0030001	metal ion transport	0.005991
GO:0098662	inorganic cation transmembrane transport	0.007187
GO:0010243	response to organonitrogen compound	0.00795
GO:0034762	regulation of transmembrane transport	0.00795
GO:0098771	inorganic ion homeostasis	0.00795
GO:0007204	positive regulation of cytosolic calcium ion concentration	0.008076
GO:0007218	neuropeptide signaling pathway	0.008076
GO:0055085	transmembrane transport	0.008076
GO:0009410	response to xenobiotic stimulus	0.008076
GO:0098660	inorganic ion transmembrane transport	0.008676
GO:0021756	striatum development	0.009231

GO:0006812	cation transport	0.010676
GO:0055080	cation homeostasis	0.010784
GO:0055065	metal ion homeostasis	0.01163
GO:0008015	blood circulation	0.01163
GO:0003014	renal system process	0.012164
GO:0033555	multicellular organismal response to stress	0.012256
GO:0042493	response to drug	0.012256
GO:0050877	nervous system process	0.012431
GO:0051966	regulation of synaptic transmission, glutamatergic	0.012796
GO:0003013	circulatory system process	0.013349
GO:0007193	adenylate cyclase-inhibiting G protein-coupled receptor signaling pathway	0.013663
GO:0051480	regulation of cytosolic calcium ion concentration	0.013736
GO:0048878	chemical homeostasis	0.014625
GO:0051049	regulation of transport	0.018787
GO:0019233	sensory perception of pain	0.020031
GO:0034220	ion transmembrane transport	0.020031
GO:0035813	regulation of renal sodium excretion	0.020416
GO:0034764	positive regulation of transmembrane transport	0.022939
GO:0009314	response to radiation	0.022939
GO:0035812	renal sodium excretion	0.022939
GO:0021544	subpallium development	0.022939
GO:0007613	memory	0.022939
GO:0007188	adenylate cyclase-modulating G protein-coupled receptor signaling pathway	0.022939
GO:0009628	response to abiotic stimulus	0.023344
GO:1901698	response to nitrogen compound	0.023904
GO:0071804	cellular potassium ion transport	0.023904
GO:0071805	potassium ion transmembrane transport	0.023904
GO:0008542	visual learning	0.024876
GO:1901381	positive regulation of potassium ion transmembrane transport	0.025185
GO:0006875	cellular metal ion homeostasis	0.028207
GO:0006873	cellular ion homeostasis	0.028207
GO:0060359	response to ammonium ion	0.028207
GO:0006813	potassium ion transport	0.028234
GO:0007632	visual behavior	0.028234
GO:0042127	regulation of cell proliferation	0.02978
GO:0043266	regulation of potassium ion transport	0.02978
GO:0007155	cell adhesion	0.02978
GO:1901379	regulation of potassium ion transmembrane transport	0.030286
GO:0032412	regulation of ion transmembrane transporter activity	0.031411
GO:0007186	G protein-coupled receptor signaling pathway	0.03182
GO:0022610	biological adhesion	0.03432
GO:0044062	regulation of excretion	0.035015
GO:0007187	G protein-coupled receptor signaling pathway, coupled to cyclic nucleotide second messenger	0.036021
GO:0050795	regulation of behavior	0.036021
GO:0006811	ion transport	0.037154
GO:0030003	cellular cation homeostasis	0.039364

GO:2001023	regulation of response to drug	0.039889
GO:0022898	regulation of transmembrane transporter activity	0.039889
GO:0051954	positive regulation of amine transport	0.041225
GO:0007200	phospholipase C-activating G protein-coupled receptor signaling pathway	0.041747
GO:0015672	monovalent inorganic cation transport	0.041747
GO:0002430	complement receptor mediated signaling pathway	0.041747
GO:0001963	synaptic transmission, dopaminergic	0.041747
GO:0043278	response to morphine	0.041747
GO:0055078	sodium ion homeostasis	0.042815
GO:0042755	eating behavior	0.042815
GO:0045471	response to ethanol	0.044009
GO:0030534	adult behavior	0.045379
GO:0014072	response to isoquinoline alkaloid	0.045379
GO:0008217	regulation of blood pressure	0.045379
GO:0006874	cellular calcium ion homeostasis	0.045379
GO:0048148	behavioral response to cocaine	0.045379
GO:0032409	regulation of transporter activity	0.048723
GO:0019932	second-messenger-mediated signaling	0.048723
GO:0043268	positive regulation of potassium ion transport	0.048723
GO:0048265	response to pain	0.049478
GO:0009607	response to biotic stimulus	0.04953

Biological Process Enrichment: 2 months (Down-regulated DEGs)

No significant results are found under threshold fdr 0.05.

Biological Process Enrichment: 7 months (Up-regulated DEGs)

GO ID:	Description	FDR (Benjamini-Hochberg)
GO:0050801	ion homeostasis	0.01891754
GO:0051480	regulation of cytosolic calcium ion concentration	0.039347211
GO:0048878	chemical homeostasis	0.039347211
GO:0006875	cellular metal ion homeostasis	0.039347211
GO:0098771	inorganic ion homeostasis	0.039347211
GO:0006873	cellular ion homeostasis	0.039347211
GO:0007204	positive regulation of cytosolic calcium ion concentration	0.039347211
GO:0072503	cellular divalent inorganic cation homeostasis	0.039347211
GO:0006874	cellular calcium ion homeostasis	0.040077615
GO:0055065	metal ion homeostasis	0.040077615
GO:0055080	cation homeostasis	0.040077615
GO:0030003	cellular cation homeostasis	0.040077615
GO:0055074	calcium ion homeostasis	0.047578647

Biological Process Enrichment: 7 months (Down-regulated DEGs)

GO ID:	Description	FDR (Benjamini-Hochberg)
GO:0034220	ion transmembrane transport	0.002177017

GO:0002476	antigen processing and presentation of endogenous peptide antigen via MHC class Ib	0.002177017
GO:0002428	antigen processing and presentation of peptide antigen via MHC class Ib	0.002177017
GO:0002475	antigen processing and presentation via MHC class Ib	0.004619368
GO:0002483	antigen processing and presentation of endogenous peptide antigen	0.004619368
GO:0019883	antigen processing and presentation of endogenous antigen	0.008558855
GO:0098655	cation transmembrane transport	0.012796569
GO:0007612	learning	0.012796569
GO:0001916	positive regulation of T cell mediated cytotoxicity	0.02057468
GO:0008306	associative learning	0.02057468
GO:0048002	antigen processing and presentation of peptide antigen	0.021769262
GO:0055085	transmembrane transport	0.037199197
GO:0001914	regulation of T cell mediated cytotoxicity	0.037199197
GO:0006811	ion transport	0.042548891
GO:0042391	regulation of membrane potential	0.043226595

Biological Process Enrichment: 8 months (Up-regulated DEGs)

GO ID:	Description	FDR (Benjamini-Hochberg)
GO:0052696	flavonoid glucuronidation	8.04E-06
GO:0052697	xenobiotic glucuronidation	8.04E-06
GO:0052695	cellular glucuronidation	8.26E-05
GO:0009812	flavonoid metabolic process	8.26E-05
GO:0006063	uronic acid metabolic process	8.26E-05
GO:0019585	glucuronate metabolic process	8.26E-05
GO:0034754	cellular hormone metabolic process	0.001763798
GO:0032787	monocarboxylic acid metabolic process	0.001763798
GO:0042981	regulation of apoptotic process	0.001763798
GO:0002138	retinoic acid biosynthetic process	0.001906694
GO:0016102	diterpenoid biosynthetic process	0.001906694
GO:0043067	regulation of programmed cell death	0.001935441
GO:0010942	positive regulation of cell death	0.002755137
GO:0043068	positive regulation of programmed cell death	0.00350972
GO:0010941	regulation of cell death	0.005631776
GO:0072330	monocarboxylic acid biosynthetic process	0.006137107
GO:0008610	lipid biosynthetic process	0.006137107
GO:0043065	positive regulation of apoptotic process	0.006137107
GO:0006915	apoptotic process	0.006137107
GO:0016114	terpenoid biosynthetic process	0.007536692
GO:0042180	cellular ketone metabolic process	0.007956559
GO:0008219	cell death	0.008439975
GO:0010565	regulation of cellular ketone metabolic process	0.008986184
GO:0012501	programmed cell death	0.008986184
GO:0006952	defense response	0.01173312
GO:0006955	immune response	0.013022822
GO:0043436	oxoacid metabolic process	0.013022822
GO:0002682	regulation of immune system process	0.014356154
GO:0042573	retinoic acid metabolic process	0.015989502

GO:0009607	response to biotic stimulus	0.016091807
GO:0051707	response to other organism	0.016091807
GO:0007611	learning or memory	0.016198472
GO:0043207	response to external biotic stimulus	0.016198472
GO:0009628	response to abiotic stimulus	0.018270087
GO:0006082	organic acid metabolic process	0.018270087
GO:0019752	carboxylic acid metabolic process	0.018270087
GO:0042445	hormone metabolic process	0.018270087
GO:0006633	fatty acid biosynthetic process	0.018270087
GO:0097529	myeloid leukocyte migration	0.018270087
GO:0006629	lipid metabolic process	0.020673448
GO:1901700	response to oxygen-containing compound	0.022922274
GO:0050801	ion homeostasis	0.025888483
GO:0046890	regulation of lipid biosynthetic process	0.025888483
GO:0060326	cell chemotaxis	0.027997706
GO:0002684	positive regulation of immune system process	0.028390743
GO:0007613	memory	0.029447632
GO:0016477	cell migration	0.032434065
GO:0014002	astrocyte development	0.033125377
GO:0030595	leukocyte chemotaxis	0.033125377
GO:0051240	positive regulation of multicellular organismal process	0.033125377
GO:0050890	cognition	0.033125377
GO:0050900	leukocyte migration	0.033125377
GO:0042127	regulation of cell proliferation	0.036760268
GO:0009416	response to light stimulus	0.03739405
GO:0046394	carboxylic acid biosynthetic process	0.038078786
GO:0016053	organic acid biosynthetic process	0.040141953
GO:0033687	osteoblast proliferation	0.041348728
GO:0042904	9-cis-retinoic acid biosynthetic process	0.041348728
GO:0042905	9-cis-retinoic acid metabolic process	0.041348728
GO:0042592	homeostatic process	0.042640654
GO:0008299	isoprenoid biosynthetic process	0.045836195

Biological Process Enrichment: 8 months (Down-regulated DEGs)

GO ID:	Description	FDR (Benjamini-Hochberg)
GO:0007610	behavior	9.85E-06
GO:0007612	learning	3.62E-04
GO:0007611	learning or memory	6.09E-04
GO:0008306	associative learning	6.09E-04
GO:0050877	nervous system process	6.09E-04
GO:0007626	locomotory behavior	6.56E-04
GO:0003008	system process	6.56E-04
GO:0050890	cognition	0.00159
GO:0015850	organic hydroxy compound transport	0.00159
GO:0015872	dopamine transport	0.001956
GO:0051937	catecholamine transport	0.002505
GO:0019933	cAMP-mediated signaling	0.003005
GO:0009719	response to endogenous stimulus	0.003716
GO:0007623	circadian rhythm	0.003716

GO:0007631	feeding behavior	0.004388
GO:0007186	G protein-coupled receptor signaling pathway	0.005412
GO:0030534	adult behavior	0.005412
GO:0007268	chemical synaptic transmission	0.005412
GO:0098916	anterograde trans-synaptic signaling	0.005412
GO:0045761	regulation of adenylate cyclase activity	0.006029
GO:0099537	trans-synaptic signaling	0.006222
GO:0015844	monoamine transport	0.006359
GO:0001975	response to amphetamine	0.006402
GO:0007218	neuropeptide signaling pathway	0.006402
GO:0007267	cell-cell signaling	0.006402
GO:0099536	synaptic signaling	0.006402
GO:0031279	regulation of cyclase activity	0.006402
GO:0007188	adenylate cyclase-modulating G protein-coupled receptor signaling pathway	0.007583
GO:0090494	dopamine uptake	0.007735
GO:0007189	adenylate cyclase-activating G protein-coupled receptor signaling pathway	0.007735
GO:0048511	rhythmic process	0.007735
GO:0019935	cyclic-nucleotide-mediated signaling	0.00865
GO:0090493	catecholamine uptake	0.010046
GO:0042752	regulation of circadian rhythm	0.011023
GO:0009725	response to hormone	0.012448
GO:0035249	synaptic transmission, glutamatergic	0.014493
GO:0006811	ion transport	0.014493
GO:0007187	G protein-coupled receptor signaling pathway, coupled to cyclic nucleotide second messenger	0.014531
GO:0006812	cation transport	0.015931
GO:0015893	drug transport	0.021747
GO:0007600	sensory perception	0.021747
GO:0030001	metal ion transport	0.02347
GO:0050953	sensory perception of light stimulus	0.023536
GO:0097305	response to alcohol	0.023536
GO:1901381	positive regulation of potassium ion transmembrane transport	0.026454
GO:0006836	neurotransmitter transport	0.026454
GO:0010737	protein kinase A signaling	0.0272
GO:0015696	ammonium transport	0.027222
GO:0071495	cellular response to endogenous stimulus	0.027222
GO:0050905	neuromuscular process	0.028812
GO:0051339	regulation of lyase activity	0.031912
GO:0001963	synaptic transmission, dopaminergic	0.03439
GO:0001964	startle response	0.03439
GO:0014075	response to amine	0.03439
GO:0023061	signal release	0.03439
GO:0034220	ion transmembrane transport	0.03439
GO:0042493	response to drug	0.036955
GO:0021879	forebrain neuron differentiation	0.039264
GO:0010738	regulation of protein kinase A signaling	0.042017
GO:0009582	detection of abiotic stimulus	0.045247

GO:0042391	regulation of membrane potential	0.045247
GO:0009581	detection of external stimulus	0.046625
GO:0007601	visual perception	0.046625
GO:0007194	negative regulation of adenylate cyclase activity	0.046625
GO:0014821	phasic smooth muscle contraction	0.047134
GO:0032228	regulation of synaptic transmission, GABAergic	0.047134

Biological Process Enrichment: 11 months (Up-regulated DEGs)

No significant results are found under threshold fdr 0.05.

Biological Process Enrichment: 11 months (Down-regulated DEGs)

GO ID:	Description	FDR (Benjamini-Hochberg)
GO:0007610	behavior	9.85E-06
GO:0007612	learning	3.62E-04
GO:0007611	learning or memory	6.09E-04
GO:0008306	associative learning	6.09E-04
GO:0050877	nervous system process	6.09E-04
GO:0007626	locomotory behavior	6.56E-04
GO:0003008	system process	6.56E-04
GO:0050890	cognition	0.001590122
GO:0015850	organic hydroxy compound transport	0.001590122
GO:0015872	dopamine transport	0.001956433
GO:0051937	catecholamine transport	0.002504656
GO:0019933	cAMP-mediated signaling	0.003004568
GO:0009719	response to endogenous stimulus	0.003715997
GO:0007623	circadian rhythm	0.003715997
GO:0007631	feeding behavior	0.004388417
GO:0007186	G protein-coupled receptor signaling pathway	0.005412379
GO:0030534	adult behavior	0.005412379
GO:0007268	chemical synaptic transmission	0.005412379
GO:0098916	anterograde trans-synaptic signaling	0.005412379
GO:0045761	regulation of adenylate cyclase activity	0.006028513
GO:0099537	trans-synaptic signaling	0.006221615
GO:0015844	monoamine transport	0.006358796
GO:0001975	response to amphetamine	0.006401764
GO:0007218	neuropeptide signaling pathway	0.006401764
GO:0007267	cell-cell signaling	0.006401764
GO:0099536	synaptic signaling	0.006401764
GO:0031279	regulation of cyclase activity	0.006401764
GO:0007188	adenylate cyclase-modulating G protein-coupled receptor signaling pathway	0.007583384
GO:0090494	dopamine uptake	0.007735068
GO:0007189	adenylate cyclase-activating G protein-coupled receptor signaling pathway	0.007735068
GO:0048511	rhythmic process	0.007735068
GO:0019935	cyclic-nucleotide-mediated signaling	0.00865032
GO:0090493	catecholamine uptake	0.010045849
GO:0042752	regulation of circadian rhythm	0.011023046

GO:0009725	response to hormone	0.012448166
GO:0035249	synaptic transmission, glutamatergic	0.014493097
GO:0006811	ion transport	0.014493097
GO:0007187	G protein-coupled receptor signaling pathway, coupled to cyclic nucleotide second messenger	0.014531219
GO:0006812	cation transport	0.015931328
GO:0015893	drug transport	0.021747097
GO:0007600	sensory perception	0.021747097
GO:0030001	metal ion transport	0.023469534
GO:0050953	sensory perception of light stimulus	0.023536146
GO:0097305	response to alcohol	0.023536146
GO:1901381	positive regulation of potassium ion transmembrane transport	0.026453659
GO:0006836	neurotransmitter transport	0.026453659
GO:0010737	protein kinase A signaling	0.027199887
GO:0015696	ammonium transport	0.027222291
GO:0071495	cellular response to endogenous stimulus	0.027222291
GO:0050905	neuromuscular process	0.028811548
GO:0051339	regulation of lyase activity	0.031912037
GO:0001963	synaptic transmission, dopaminergic	0.034389501
GO:0001964	startle response	0.034389501
GO:0014075	response to amine	0.034389501
GO:0023061	signal release	0.034389501
GO:0034220	ion transmembrane transport	0.034389501
GO:0042493	response to drug	0.036954803
GO:0021879	forebrain neuron differentiation	0.039263775
GO:0010738	regulation of protein kinase A signaling	0.042016684
GO:0009582	detection of abiotic stimulus	0.045246768
GO:0042391	regulation of membrane potential	0.045246768
GO:0009581	detection of external stimulus	0.046624612
GO:0007601	visual perception	0.046624612
GO:0007194	negative regulation of adenylate cyclase activity	0.046624612
GO:0014821	phasic smooth muscle contraction	0.047133881
GO:0032228	regulation of synaptic transmission, GABAergic	0.047133881

Biological Process Enrichment: 14 months (Up-regulated DEGs)

No significant results are found under threshold fdr 0.05.

Biological Process Enrichment: 14 months (Down-regulated DEGs)

GO ID:	Description	FDR (Benjamini-Hochberg)
GO:0051321	meiotic cell cycle	3.21E-02
GO:0007127	meiosis I	3.21E-02
GO:0061982	meiosis I cell cycle process	3.21E-02
GO:0140013	meiotic nuclear division	3.21E-02
GO:1903046	meiotic cell cycle process	4.88E-02
